# Supplementary material for: Proteome remodelling during development from blood to insect-form Trypanosoma brucei quantified by SILAC and mass spectrometry
Source: BMC Genomics. 2012 Oct 16;13:556. doi: 10.1186/1471-2164-13-556 (PMC3545838; doi:10.1186/1471-2164-13-556)
Supplement: Additional file 1 — Figure S1. Comparison of procyclic T. brucei cell growth in different media. (A) Cells grown in SDM 79/80 with regular FCS and SDM 80 with dialyzed FCS (dFCS). (B) Comparison of procyclic T. brucei cell growth of cells in SDM 80 dFCS regular amino acids and cells in SDM 80, dFCS and heavy isotope labeled amino acids (Arg; Lys). Figure S2. Labelling efficiency of procyclic T. brucei whole cell proteome after zero, two and 11 cell division cycles. (A) Box plot depicting mean ratios of heavy/light peptides from the whole proteome. (B) Shows the relative abundance shift of mass spectrometry peaks (m/z) during the course of labelling (0, 2 and 11 cell division cycles) with heavy amino acids towards higher m/z ratios. Data corresponds to one peptide of the triosephosphate isomerase gene (Tb11.02.3210). Figure S3. Expression profile of 166 mitochondrial proteins during T. brucei development. Each circle indicates a cluster of co-regulated proteins. Circle size is proportional to the number of proteins in the cluster. The midpoint of the circle marks the mean fold change between the life cycle stages. Expression profile is depicted as fold change (log2) between the different life cycle stages. Figure S4. Protein expression profile of oxidative phosphorylation complexes I-V during development. (A and B) Complex one. (C and D) Complex two. (E and F) Complex three. (G and H) Complex four. (I and J) Complex five. Each gene is assigned a sign. x-axis indicate the fold regulation in log2 between life stages. Figure S5. Procyclic specific metabolism genes and their regulation during development. (A) Abundance profile of eight different proteins likely involved in pyruvate to acetate conversion in the mitochondrion of insect form trypanosomes. (B) Model depicting the conversion of pyruvate to acetate. Green arrow indicates activation of the pyruvate dehydrogenase complex through dephosphorylation. Figure S6. Gene expression comparison between SLT, microarray and MS/MS. (A) A set of [file 1471-2164-13-556-S1.pdf]

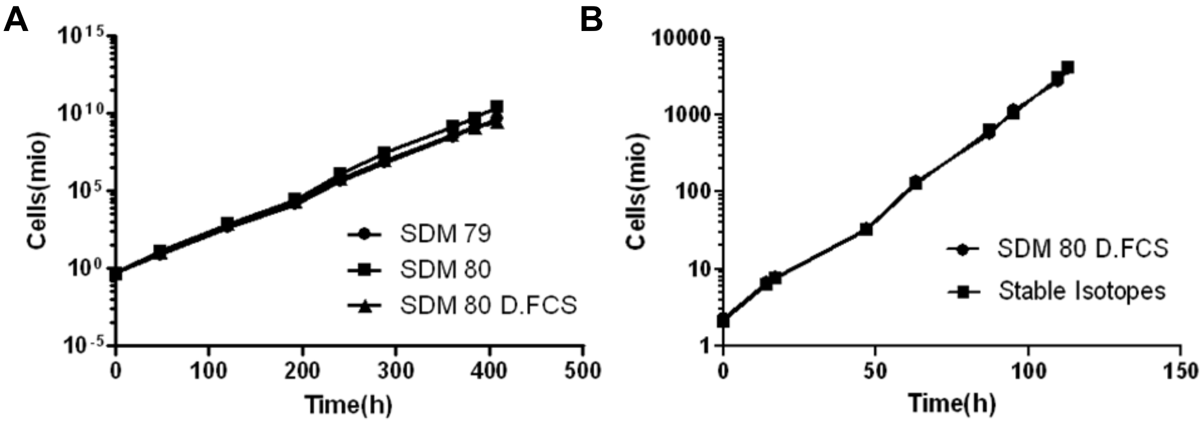

Figure S1: Comparison of procyclic *T. brucei* cell growth in different media. **(A)** Cells grown in SDM 79/80 with regular FCS and SDM 80 with dialyzed FCS (dFCS). **(B)** Comparison of procyclic *T. brucei* cell growth of cells in SDM 80 dFCS regular amino acids and cells in SDM 80, dFCS and heavy isotope labeled amino acids (Arg; Lys).

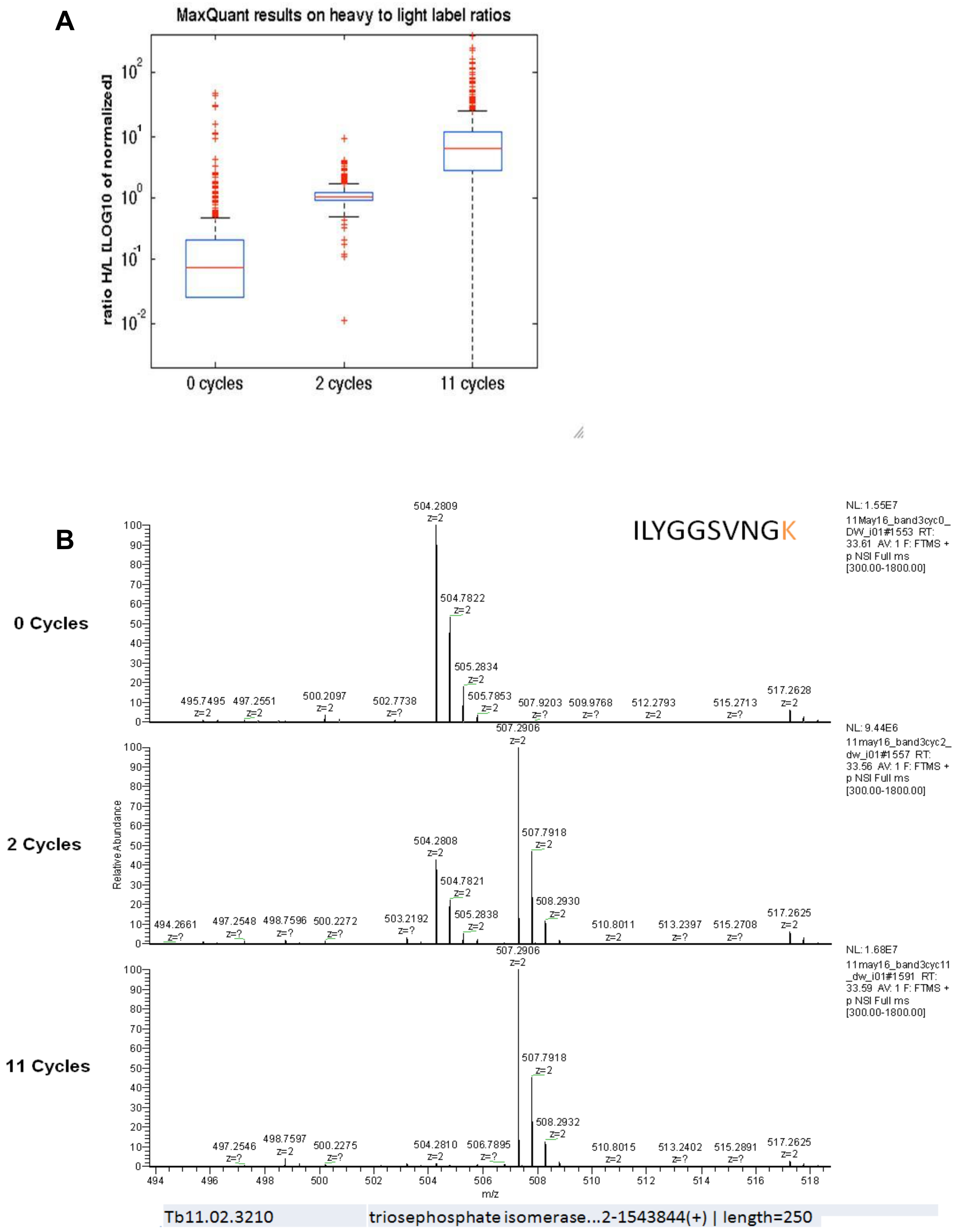

Figure S2: Labelling efficiency of procyclic *T. brucei* whole cell proteome after zero, two and 11 cell division cycles. **(A)** Box plot depicting mean-ratios of heavy/light peptides from the whole proteome. **(B)** Shows the relative abundance shift of mass spectrometry peaks (m/z) during the course of labelling (0, 2 and 11 cell division cycles) with heavy amino acids towards higher m/z ratios. Data corresponds to one peptide of the triosephosphate isomerase gene (Tb11.02.3210).

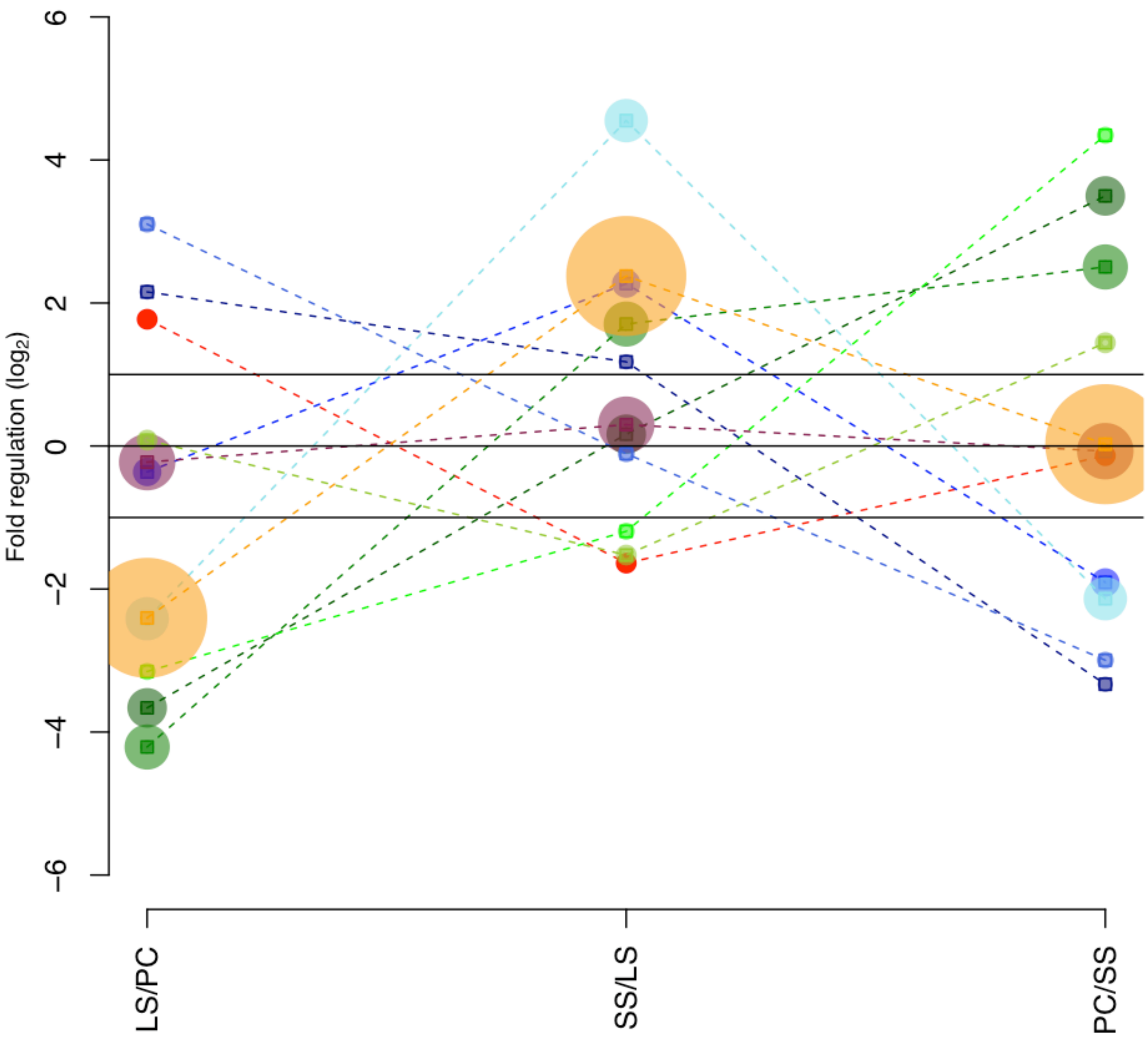

Figure S3: Expression profile of 166 mitochondrial proteins during *T. brucei* development. Each circle indicates a cluster of co-regulated proteins. Circle size is proportional to the number of proteins in the cluster. The midpoint of the circle marks the mean fold change between the life cycle stages. Expression profile is depicted as fold change (log_2) between the different life cycle stages.

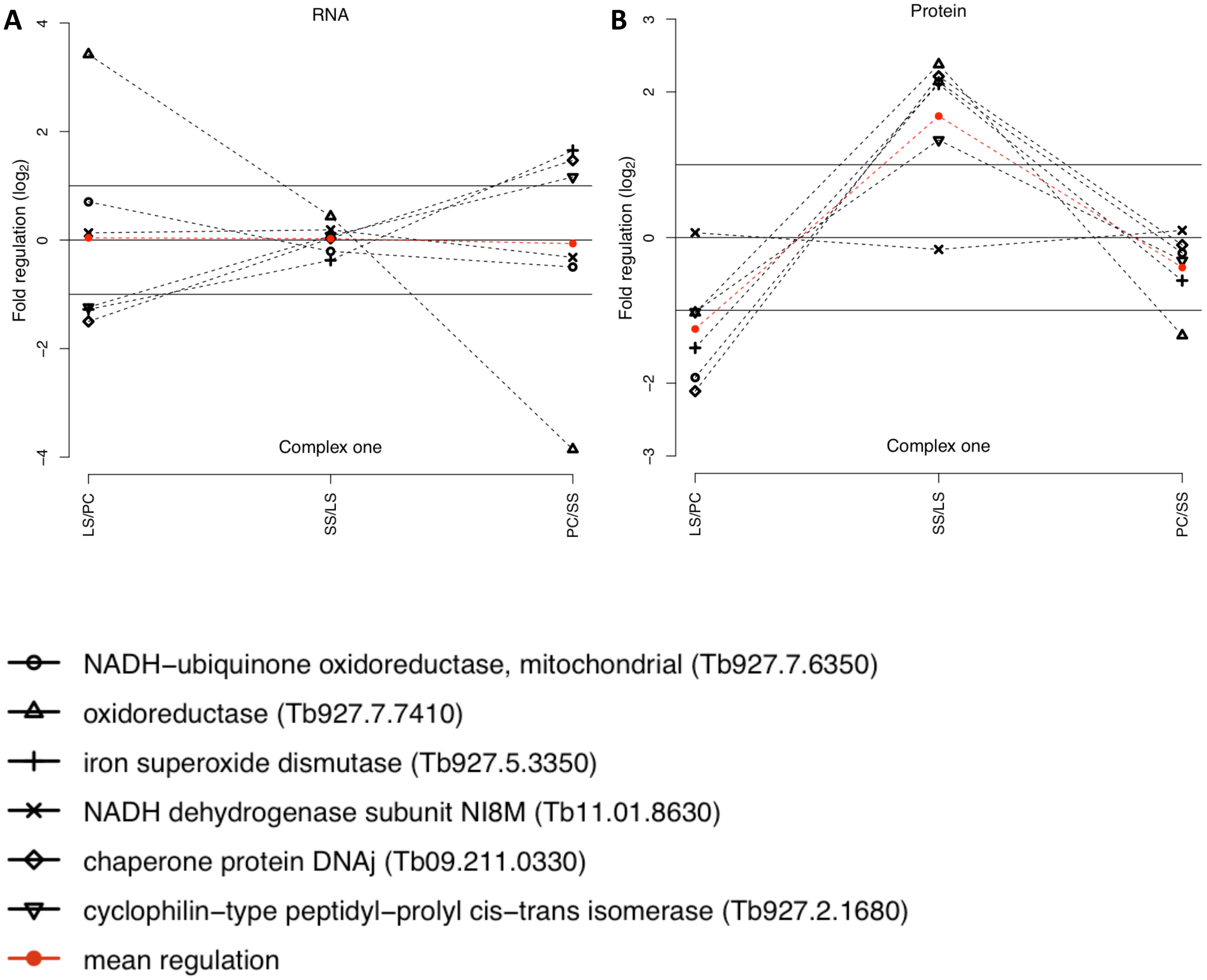
A
RNA
Fold regulation (log2)
4
2
0
−2
−4
Complex one
LS/PC
SS/LS
PC/SS
B
Protein
Fold regulation (log2)
3
2
0
−2
−3
Complex one
LS/PC
SS/LS
PC/SS
NADH−ubiquinone oxidoreductase, mitochondrial (Tb927.7.6350)
oxidoreductase (Tb927.7.7410)
iron superoxide dismutase (Tb927.5.3350)
NADH dehydrogenase subunit NI8M (Tb11.01.8630)
chaperone protein DNAj (Tb09.211.0330)
cyclophilin−type peptidyl−prolyl cis−trans isomerase (Tb927.2.1680)
mean regulation

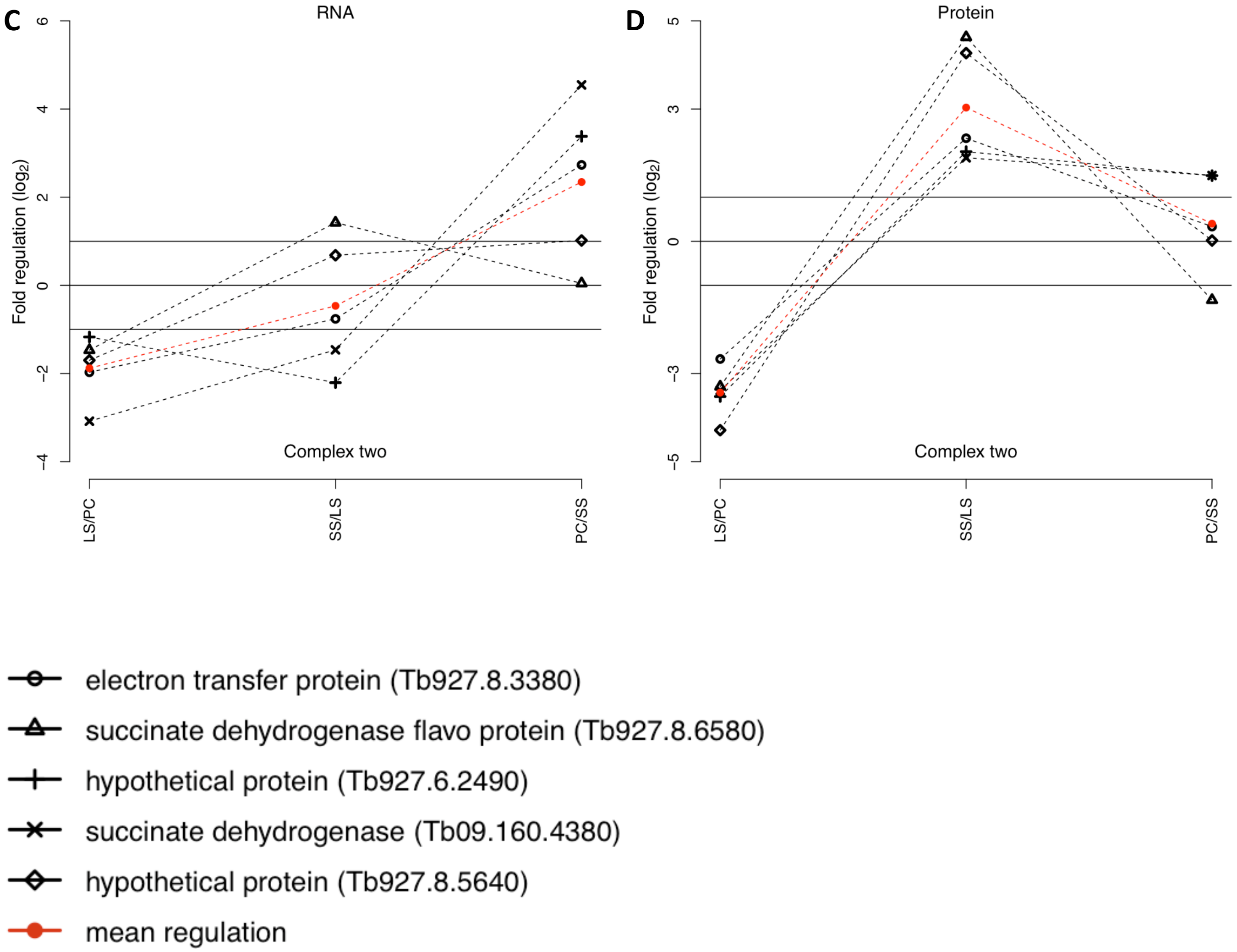
C
RNA
Fold regulation (log2)
Complex two
LS/PC
SS/LS
PC/SS
D
Protein
Fold regulation (log2)
Complex two
LS/PC
SS/LS
PC/SS
electron transfer protein (Tb927.8.3380)
succinate dehydrogenase flavo protein (Tb927.8.6580)
hypothetical protein (Tb927.6.2490)
succinate dehydrogenase (Tb09.160.4380)
hypothetical protein (Tb927.8.5640)
mean regulation

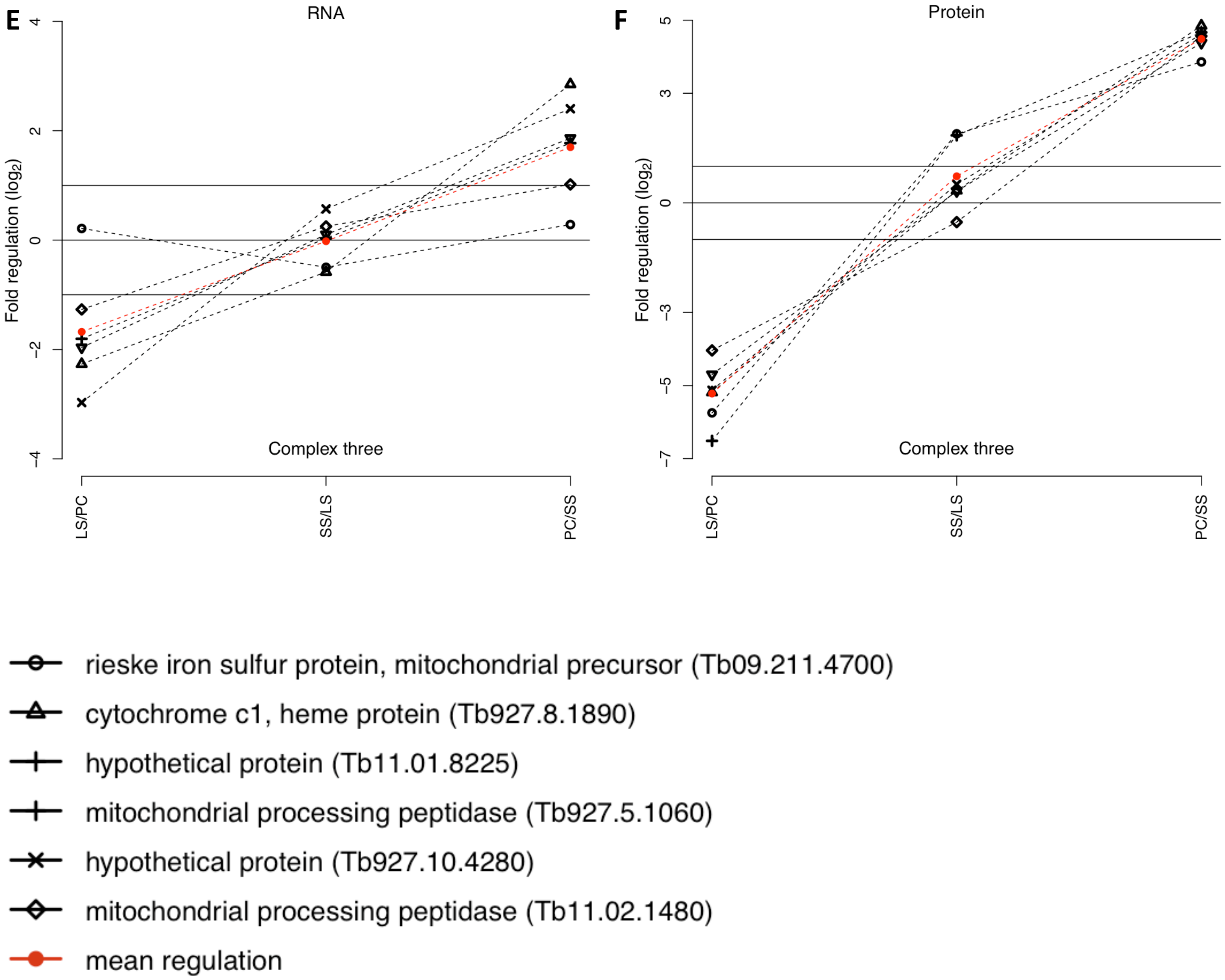

E
RNA
Fold regulation (log2)
4
2
0
−2
−4
Complex three
LS/PC
SS/LS
PC/SS
F
Protein
Fold regulation (log2)
5
3
0
−3
−5
−7
Complex three
LS/PC
SS/LS
PC/SS
rieske iron sulfur protein, mitochondrial precursor (Tb09.211.4700)
cytochrome c1, heme protein (Tb927.8.1890)
hypothetical protein (Tb11.01.8225)
mitochondrial processing peptidase (Tb927.5.1060)
hypothetical protein (Tb927.10.4280)
mitochondrial processing peptidase (Tb11.02.1480)
mean regulation

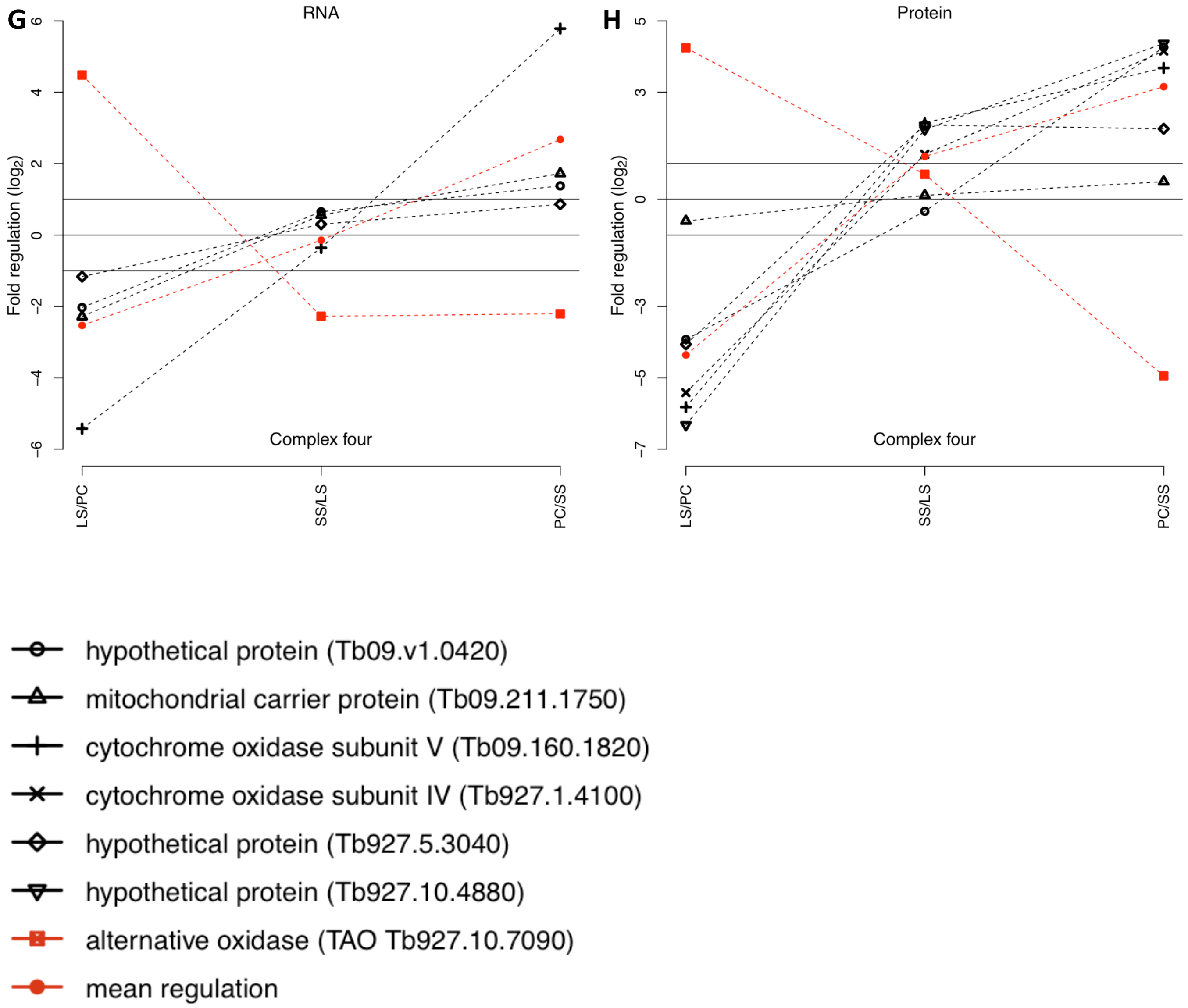
G
RNA
Fold regulation (log2)
6
4
2
0
−2
−4
−6
Complex four
LS/PC
SS/LS
PC/SS
H
Protein
Fold regulation (log2)
5
3
0
−3
−5
−7
Complex four
LS/PC
SS/LS
PC/SS
hypothetical protein (Tb09.v1.0420)
mitochondrial carrier protein (Tb09.211.1750)
cytochrome oxidase subunit V (Tb09.160.1820)
cytochrome oxidase subunit IV (Tb927.1.4100)
hypothetical protein (Tb927.5.3040)
hypothetical protein (Tb927.10.4880)
alternative oxidase (TAO Tb927.10.7090)
mean regulation

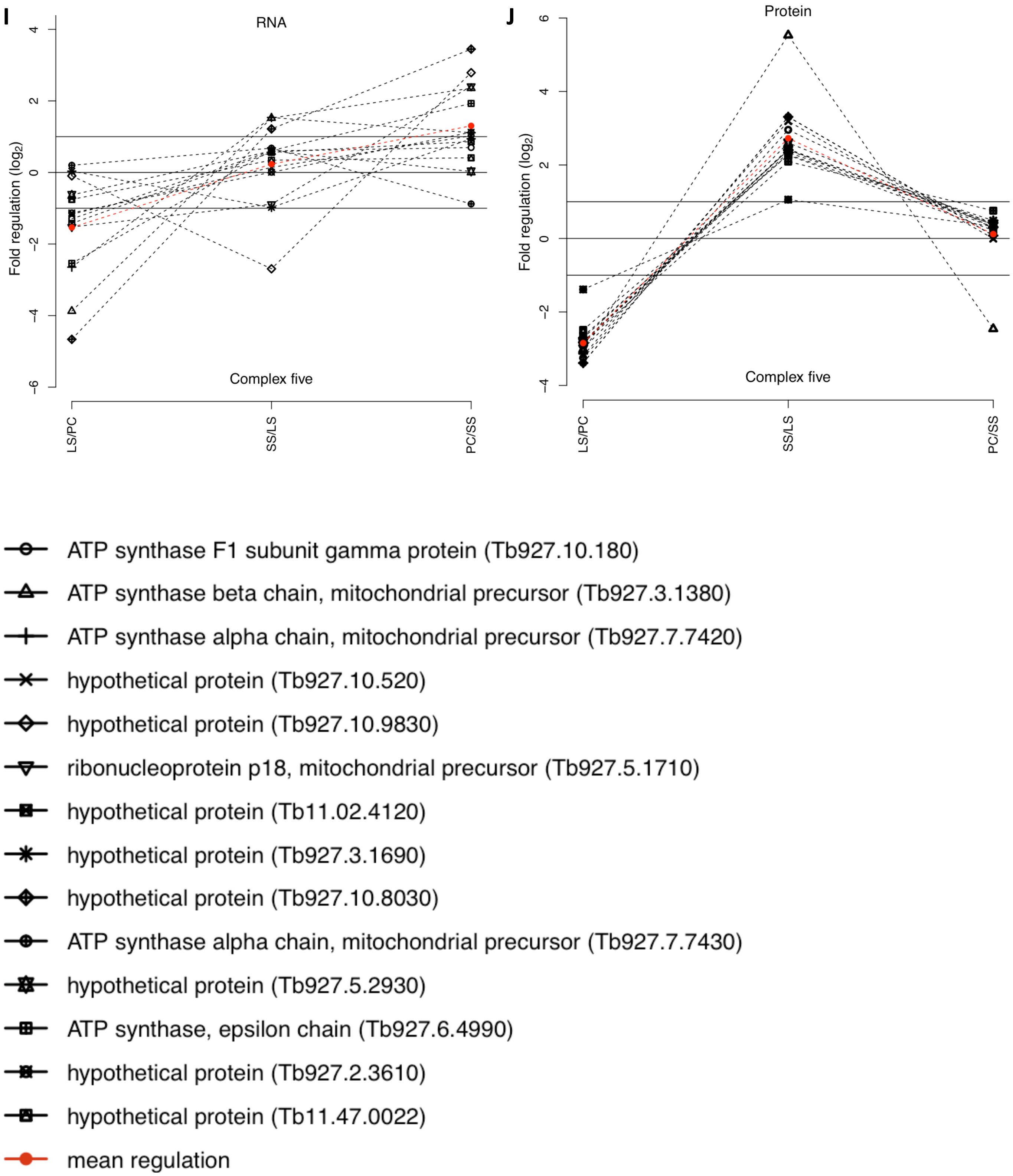

Figure S4: Protein expression profile of oxidative phosphorylation complexes I-V during development. **(A and B)** Complex one. **(C and D)** Complex two. **(E and F)** Complex three. **(G and H)** Complex four. **(I and J)** Complex five. Each gene is assigned a sign. x-axis indicate the fold regulation in log_2 between life stages.

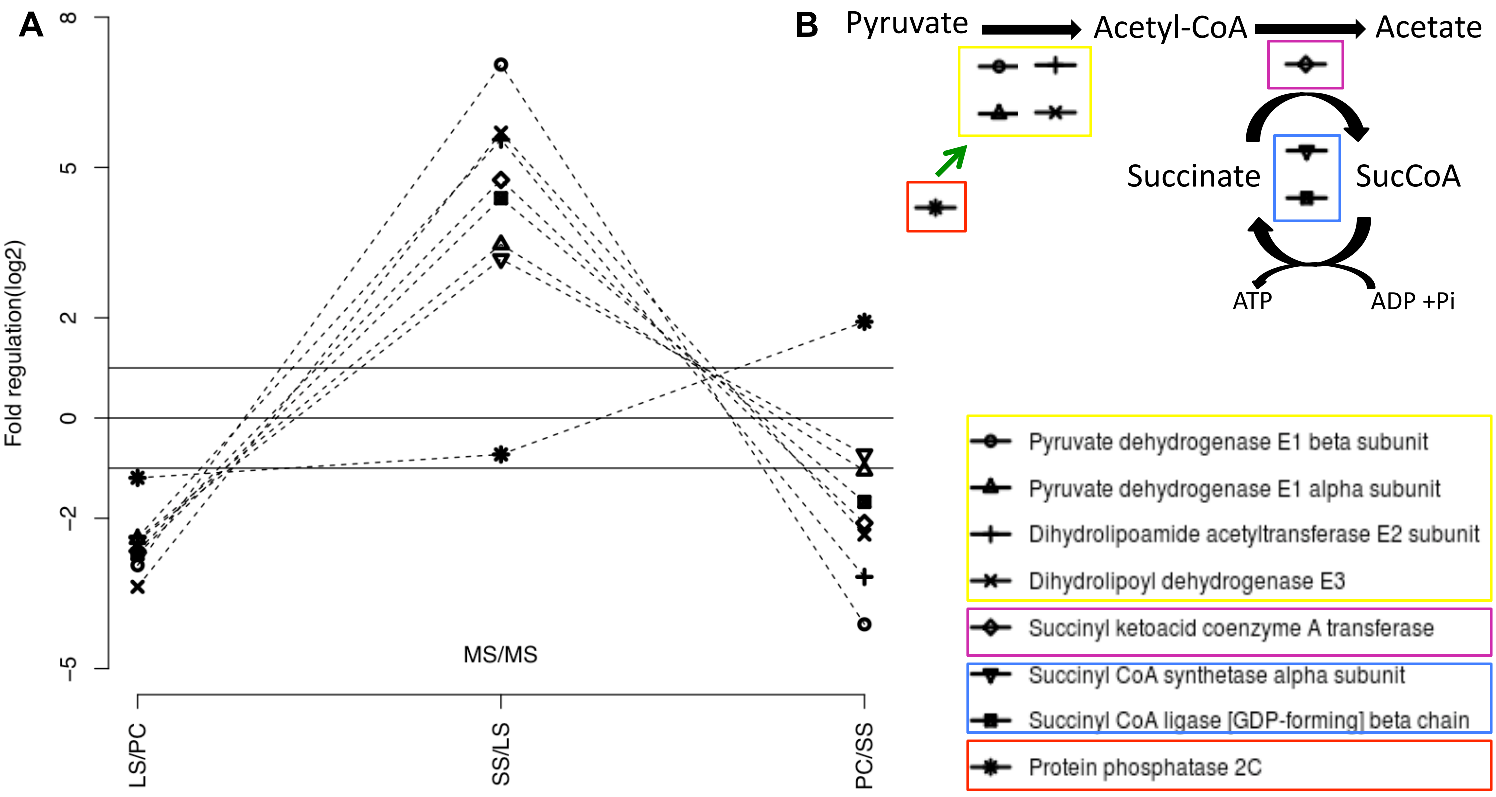

Figure S5: Procyclic specific metabolism genes and their regulation during development. **(A)** Abundance profile of eight different proteins likely involved in pyruvate to acetate conversion in the mitochondrion of insect form trypanosomes. **(B)** Model depicting the conversion of pyruvate to acetate. Green arrow indicates activation of the pyruvate dehydrogenase complex through dephosphorylation.

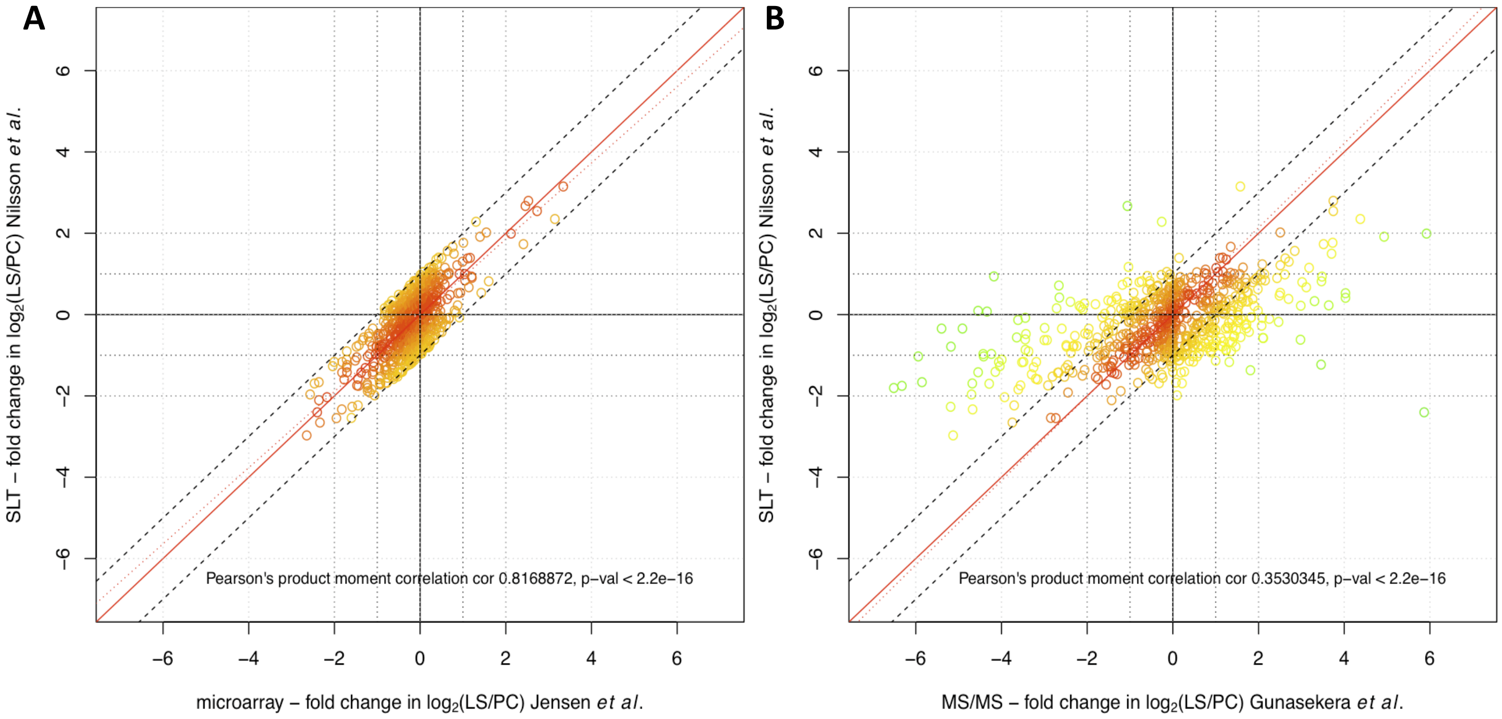

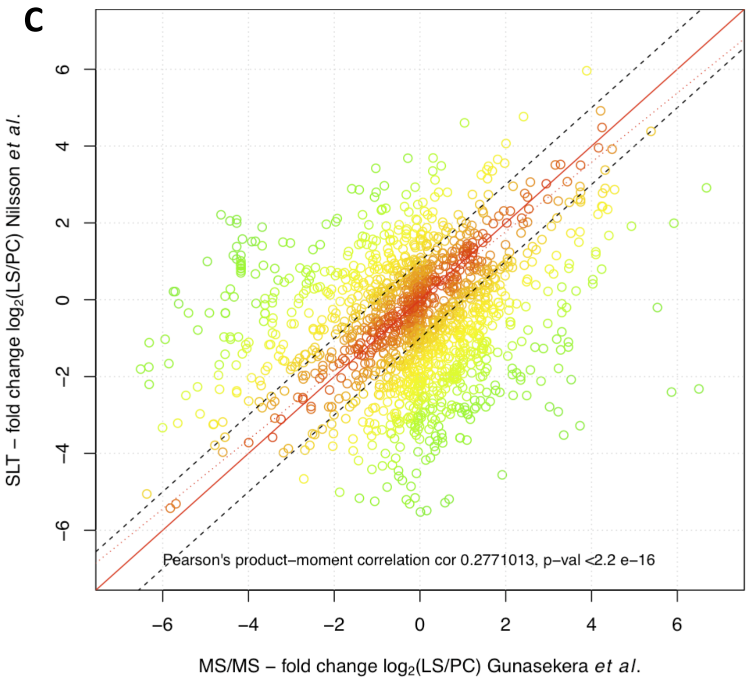

Figure S6: Gene expression comparison between SLT, microarray and MS/MS. **(A)** A set of highly correlated genes between microarray (Jensen *et al*) and SLT (Nilsson *et al*). The Pearson's product moment correlation is 0.82 (p-value < 2.2e-16). **(B)** Correlation of the same set between SLT and MS/MS. The Pearson's product moment correlation is 0.35 (p-value < 2.2e-16). **(C)** Correlation of mRNA and protein changes between LS and PC. The Pearson's product moment correlation is 0.28 (p-value < 2.2e-16). Colors of the circles in the scatter plot indicate correlation between the two studies (red high correlation, green low correlation).
