# Supplementary material for: Proteome remodelling during development from blood to insect-form Trypanosoma brucei quantified by SILAC and mass spectrometry
Source: BMC Genomics. 2012 Oct 16;13:556. doi: 10.1186/1471-2164-13-556 (PMC3545838; doi:10.1186/1471-2164-13-556)
Supplement: Additional file 3 — Table S1. Antibodies and conditions used in this study. [file 1471-2164-13-556-S3.pdf]

Table S1: Antibodies and conditions used in this study

Antibody	Gene ID	Animal	Cells on gel (*10^6)	Dilution	Incubation time, Temp	Provider
ALBA 3	Tb927.4.2040	Rabbit	1	1:500	15 h, 4°C	I. Roditi
ISP	Tb09 211.4700	Mouse (monoclonal)	4	1:4000	15 h, 4°C	S. Hajduk
HSP 70	Tb927.6.3740	Rabbit	4	1:2000	1 h, RT	S. Hajduk
PFR	Tb927.8.4980	Rat	1	1:1000	1 h, RT	T. Seebeck
Trypanopain	Tb927.6.560	Rabbit	1	1:4000	15 h, 4°C	J. Bangs
ATOM	Tb09.211.1240	Rabbit	4	1:20'000	1 h, RT	A. Schneider
COX IV	Tb927.1.4100	Rabbit	4	1:1000	1 h, RT	A. Schneider
Aldolase	Tb927.10.5620	Rabbit	4	1:1000	1 h, RT	C. Clayton
MRP 2	Tb11.01.4860	Rabbit	4	1:1000	1 h, RT	J. Lukes

* *Gene ID allows access to all information about the corresponding protein at GeneDB: http://www.genedb.org/Homepage*
