# Supplementary material for: Proteome remodelling during development from blood to insect-form Trypanosoma brucei quantified by SILAC and mass spectrometry
Source: BMC Genomics. 2012 Oct 16;13:556. doi: 10.1186/1471-2164-13-556 (PMC3545838; doi:10.1186/1471-2164-13-556)
Supplement: Additional file 11 — Table S10. Change of mitochondrial protein abundance during differentiation. (PDF 53 kb) [file 1471-2164-13-556-S11.pdf]

Table S10: Change of mitochondrial protein abundance during differentiation

	MS/MS				MS/MS overlaps			RNA-Seq and MS/MS overlaps		
	LS/PC	SS/LS	PC/SS	total	LS/PC	SS/LS	PC/SS	LS/PC	SS/LS	PC/SS
All detected	251		208	293	166	166	166	162	162	162
Upregulated ≥2 fold	19		41		8	111	38	8	108	37
Downregulated ≥2 fold	167		40		114	10	31	111	10	31
Unchanged in regulation	65		127		44	45	97	43	44	94
