# Supplementary material for: Proteome remodelling during development from blood to insect-form Trypanosoma brucei quantified by SILAC and mass spectrometry
Source: BMC Genomics. 2012 Oct 16;13:556. doi: 10.1186/1471-2164-13-556 (PMC3545838; doi:10.1186/1471-2164-13-556)
Supplement: Additional file 12 — Table S11. Coverage of novel transcripts. [file 1471-2164-13-556-S12.pdf]

Table S11: Coverage of novel transcripts

entry_no	protein_id	protein_prob	no_of_uniqu	peptide_sequence
1	Tb11.NT.20.1	1	2	MSFFQLDWECSYQEYMKMIYHHATNYVPPRVSQER
2	Tb11.NT.57.1	1	1	TEWMYYHGHGGCPGKYGPSREIADFEYADGTPASISGR
2	Tb11.NT.57.1	1	3	NWDPAIPLFLDDVDEQGRPAPLRTAGDAPGTMVSHVCSRVVDER
3	Tb3.NT.43.1	1	1	SEDAYFDALKAVENSPMSADAAGSAAYPVSQNDICLCLAQQLLYRTR
3	Tb3.NT.43.1	1	1	SIQEYILSLKKPSPSQALHGTDNHASLVLAFQLSR
3	Tb3.NT.43.1	1	2	AVENSPMSADAAGSAAYPVSQNDICLCLAQQLLYR
3	Tb3.NT.43.1	1	3	DLGAALTDEEVVELVYIFEAAHIRHHFHDYFPLHEDYR
3	Tb3.NT.43.1	1	8	TSLAKSEDAYFDALKAVENSPMSADAAGSAAYPVSQNDICLCLAQQLLYR
4	Tb4.NT.20.1	1	2	GSSGFCISSLSFSPQLVAMNGDLSPSERWDDYDGYDQYNNNNINGDDDDDDDDSNCK
5	Tb4.NT.51.1	1	2	MLLSGDVEENPGPSLRGMQWNCAGLSKGGGCGCAESCAHTGPLSTNQIVVCEFPVVPDR
6	Tb5.NT.25.1	1	1	QISPFLHAACNEFAALPLIPALFFFVSALPVKPTR
6	Tb5.NT.25.1	1	1	QISPFLHAACNEFAALPLIPALFFFVSALPVKPTRKYMLK
6	Tb5.NT.25.1	1	1	VSKQISPFLHAACNEFAALPLIPALFFFVSALPVKPTR
7	Tb6.NT.63.14	1	11	YGDATPAVDILPEPSPVVAIVPLPSSIRVLMRPQVLYCTLCASKFAVPGR
7	Tb6.NT.63.14	1	6	TSEPTKCTECDATYQYLSNAVTHMVNKHGFVR
8	Tb6.NT.63.8	1	12	LLGVTFQCLQGMATHAAETRR
8	Tb6.NT.63.8	1	2	LLGVTFQCLQGMATHAAETRRK
8	Tb6.NT.63.8	1	2	TPKLLGVTFQCLQGMATHAAETRR
8	Tb6.NT.63.8	1	2	TTTLVRSMKFMLMCESR
8	Tb6.NT.63.8	1	36	IGADRTPKLLGVTFQCLQGMATHAAETR
8	Tb6.NT.63.8	1	3	EHPLETSTLRHSCRPIFHTQIKPVCADDPDNVK
8	Tb6.NT.63.8	1	4	LLGVTFQCLQGMATHAAETR
9	Tb7.NT.16.1	1	1	FSMNTVTHSPHSLGSQHNVMTASPFVTPAYVDALDHLSTKLR
9	Tb7.NT.16.1	1	1	QEALEQPTCCFFHGAPCTSSGLPLNLQVNVGGTSITLPR
9	Tb7.NT.16.1	1	6	MSFSMNTVTHSPHSLGSQHNVMTASPFVTPAYVDALDHLSTK
9	Tb7.NT.16.1	1	8	MSFSMNTVTHSPHSLGSQHNVMTASPFVTPAYVDALDHLSTKLR
10	Tb7.NT.18.1	1	10	CCGEGWKYGDHKVYWNGESGDVFVGSFIVAK
10	Tb7.NT.18.1	1	10	GCIVPHSSMEDTLSSFLDQLAELQVETMER
10	Tb7.NT.18.1	1	10	WIQPQTYETTVRCCGEGWKYGDHK
10	Tb7.NT.18.1	1	11	ARVTFGDVTVVPYAGTRPGPAGNK
10	Tb7.NT.18.1	1	11	GAEQHSKSTVETTTSLIKPTTSGYTGSESYSASK
10	Tb7.NT.18.1	1	11	GCIVPHSSMEDTLSSFLDQLAELQVETMERNRVLK
10	Tb7.NT.18.1	1	11	TNSNESSSYSQTIESSGTKTQPPPAR
10	Tb7.NT.18.1	1	12	AVEEPKGAEQHSKSTVETTTSLIKPTTSGYTGSESYSASK
10	Tb7.NT.18.1	1	12	YGDHKVYWNGESGDVFVGSFIVAKGITER
10	Tb7.NT.18.1	1	13	LKSGKLPVRPGSCYTYTPLDEALK
10	Tb7.NT.18.1	1	13	SGDYSIELSGEGGVTSSESTEIPSPARIRK
10	Tb7.NT.18.1	1	14	GCIVPHSSMEDTLSSFLDQLAELQVETMERNR
10	Tb7.NT.18.1	1	14	YIEAFLYVCHAIMVVTVGTGMK
10	Tb7.NT.18.1	1	15	RWIQPQTYETTVRCCGEGWK
10	Tb7.NT.18.1	1	15	VAVQLLRYIEAFLYVCHAIMVVTVGTGMKLR
10	Tb7.NT.18.1	1	16	AAAPARPVEPPKPETHSHTAPPAASPIVRGR
10	Tb7.NT.18.1	1	16	ARVTFGDVTVVPYAGTRPGPAGNKR
10	Tb7.NT.18.1	1	16	RPSLRTPEIPKEDQQQTHTSVQQRPANAK
10	Tb7.NT.18.1	1	17	AAAPARPVEPPKPETHSHTAPPAASPIVR
10	Tb7.NT.18.1	1	17	TPEIPKEDQQQTHTSVQQRPANAK
10	Tb7.NT.18.1	1	17	WLRQQQVQCCFMVTADSELMDILQGLWK
10	Tb7.NT.18.1	1	18	DRYLCEGDDAVLHDTIRPKHLVPK
10	Tb7.NT.18.1	1	19	TNRARVTFGDVTVVPYAGTRPGPAGNK
10	Tb7.NT.18.1	1	1	APATSTEGLKIDNFSGSGSDYYSYSPYGSHSGSYTGSYSSYSYTQR
10	Tb7.NT.18.1	1	1	APQPEQDANVVSVSSSELGYTGSYYDSEYDYSYDYSESATKTQK
10	Tb7.NT.18.1	1	1	ASQADESSSYSYSDYSDDYSYYGSYTQSSRTNSNESSSYSQTIESSGTK
10	Tb7.NT.18.1	1	1	DVPDLKTLPSRCLSLHR
10	Tb7.NT.18.1	1	1	EQQQQQQQQQQQQSYTQSGQLQPQLQVQPLAPQHPPRAPVAPQQPVPSKSVQGPR
10	Tb7.NT.18.1	1	1	GNSAARVSTESYTGSYSSYSYSYSDSTDEGHNVVASARSGHVAGAAQLDSEK
10	Tb7.NT.18.1	1	1	GVEGCYLEVGVEKLRISEK
10	Tb7.NT.18.1	1	1	HPLPPSHVVKAPQPEQDANVVSVSSSELGYTGSYYDSEYDYSYDYSESATKTQK
10	Tb7.NT.18.1	1	1	IDNFSGSGSDYYSYSPYGSHSGSYTGSYSSYSYTQR
10	Tb7.NT.18.1	1	1	ILILATKVVAAHNNITGGDQGIITDVVAGK
10	Tb7.NT.18.1	1	1	ISEKVPENARCYCLVSVR
10	Tb7.NT.18.1	1	1	LLNPTVKGVTDATSFLYELAFGSVFNPKK
10	Tb7.NT.18.1	1	1	SGKLPVRPGSCYTYTPLDEALKQPVEASGGFCAPR
10	Tb7.NT.18.1	1	1	SKDSEWMTAEVLQAVSVGKANK
10	Tb7.NT.18.1	1	1	SSSDRLFFTGAIEDSLYVEACYDIVDASGETTTYCVGHVLVPCGK
10	Tb7.NT.18.1	1	1	STVETTTSLIKPTTSGYTGSESYSASKSGDYSIELSGEGGVTSSESTEIPSPAR
10	Tb7.NT.18.1	1	1	TILEYYGSRCLTEKVMSMFVAYDEFYSGEVPLSIAFEILEYCGLVTQTVGK
10	Tb7.NT.18.1	1	1	TQPPPAREQQQQQQQQQQQQSYTQSGQLQPQLQVQPLAPQHPPR
10	Tb7.NT.18.1	1	1	VMSMFVAYDEFYSGEVPLSIAFEILEYCGLVTQTVGK
10	Tb7.NT.18.1	1	1	VYWNGESGDVFVGSFIVAKGITER
10	Tb7.NT.18.1	1	1	YQQAVVGHQHPPMAQPHLQMGVQQVSPWAQQVPVGNQQPPAVHSQPPANIQHPR
10	Tb7.NT.18.1	1	22	QQQVQCCFMVTADSELMDILQGLWKR
10	Tb7.NT.18.1	1	22	YIEAFLYVCHAIMVVTVGTGMKLRVLYTPLVEK
10	Tb7.NT.18.1	1	24	VAVQLLRYIEAFLYVCHAIMVVTVGTGMK
10	Tb7.NT.18.1	1	26	WLRQQQVQCCFMVTADSELMDILQGLWKR
10	Tb7.NT.18.1	1	29	TLPSRCLSLHRHVSLMSLMCAAVISGR
10	Tb7.NT.18.1	1	2	AAAPARPVEPPKPETHSHTAPPAASPIVRGRVQDDDSYSYSYSYSYGSVSSPEVK
10	Tb7.NT.18.1	1	2	APQPEQDANVVSVSSSELGYTGSYYDSEYDYSYDYSESATK
10	Tb7.NT.18.1	1	2	APQPEQDANVVSVSSSELGYTGSYYDSEYDYSYDYSESATKTQKAVEEPK

10	Tb7.NT.18.1	1	2	DDELNTLLMSTANSYTTVSANIWTQKGVEGCYLEVGVEK
10	Tb7.NT.18.1	1	2	EDQQQTHTSVQQRPANAK
10	Tb7.NT.18.1	1	2	FKIFLRPQVSQGQILLCVADQPQEATENIVDDAEGEKR
10	Tb7.NT.18.1	1	2	GVTDATSFLYELAFGSVFNPK
10	Tb7.NT.18.1	1	2	HVSLMSLMCAAVISGRDGGSHPLR
10	Tb7.NT.18.1	1	2	LFFTGAIEDSLYVEACYDIVDASGETTTYCVGHVLVPCGKLK
10	Tb7.NT.18.1	1	2	LRVLYTPLVEKLLNPTVK
10	Tb7.NT.18.1	1	2	TQLGRAPATSTEGLKIDNFSGSGSDYYSYSPYGSHSGSYTGSYSSYSYTQR
10	Tb7.NT.18.1	1	2	VSTESYTGSYSSYSYSYSDSTDEGHNVVASARSGHVAGAAQLDSEK
10	Tb7.NT.18.1	1	33	VGSQQPVGMAHKYQAARTQQMPSK
10	Tb7.NT.18.1	1	3	APFMINGDKDDELNTLLMSTANSYTTVSANIWTQKGVEGCYLEVGVEK
10	Tb7.NT.18.1	1	3	DRYLCEGDDAVLHDTIRPK
10	Tb7.NT.18.1	1	3	ILILATKVVAAHNNITGGDQGIITDVVAGKK
10	Tb7.NT.18.1	1	3	KTILLAAPSEAEPRFHSLPR
10	Tb7.NT.18.1	1	3	QQQVQCCFMVTADSELMDILQGLWKRVMK
10	Tb7.NT.18.1	1	3	SGDYSIELSGEGGVTSSESTEIPSPAR
10	Tb7.NT.18.1	1	3	VTFGDVTVVPYAGTRPGPAGNK
10	Tb7.NT.18.1	1	3	VVAAHNNITGGDQGIITDVVAGKKGIPFTENMNRPLRPVCV
10	Tb7.NT.18.1	1	3	YIEAFLYVCHAIMVVTVGTGMKLR
10	Tb7.NT.18.1	1	3	YLCEGDDAVLHDTIRPKHLVPK
10	Tb7.NT.18.1	1	4	ASQADESSSYSYSDYSDDYSYYGSYTQSSR
10	Tb7.NT.18.1	1	4	DGGSHPLRWLRQQQVQCCFMVTADSELMDILQGLWK
10	Tb7.NT.18.1	1	4	EQQQQQQQQQQQQSYTQSGQLQPQLQVQPLAPQHPPR
10	Tb7.NT.18.1	1	4	EQQQQQQQQQQQQSYTQSGQLQPQLQVQPLAPQHPPRAPVAPQQPVPSK
10	Tb7.NT.18.1	1	4	GNSAARVSTESYTGSYSSYSYSYSDSTDEGHNVVASAR
10	Tb7.NT.18.1	1	4	HGDDPLMSARSRQQPPTGSR
10	Tb7.NT.18.1	1	4	SGKLPVRPGSCYTYTPLDEALK
10	Tb7.NT.18.1	1	4	SVQGPRGNSAARVSTESYTGSYSSYSYSYSDSTDEGHNVVASAR
10	Tb7.NT.18.1	1	4	VTFGDVTVVPYAGTRPGPAGNKR
10	Tb7.NT.18.1	1	4	YGDHKVYWNGESGDVFVGSFIVAK
10	Tb7.NT.18.1	1	5	GRVQDDDSYSYSYSYSYGSVSSPEVKHPLPPSHVVK
10	Tb7.NT.18.1	1	5	HLVPKAPFMINGDKDDELNTLLMSTANSYTTVSANIWTQK
10	Tb7.NT.18.1	1	5	IASDEANSPADIVEAPISVIPSRNLTRK
10	Tb7.NT.18.1	1	5	RASQADESSSYSYSDYSDDYSYYGSYTQSSR
10	Tb7.NT.18.1	1	5	RASQADESSSYSYSDYSDDYSYYGSYTQSSRTNSNESSSYSQTIESSGTK
10	Tb7.NT.18.1	1	5	SSSDRLFFTGAIEDSLYVEACYDIVDASGETTTYCVGHVLVPCGKLK
10	Tb7.NT.18.1	1	5	VQDDDSYSYSYSYSYGSVSSPEVK
10	Tb7.NT.18.1	1	5	VQDDDSYSYSYSYSYGSVSSPEVKHPLPPSHVVK
10	Tb7.NT.18.1	1	5	VSTESYTGSYSSYSYSYSDSTDEGHNVVASAR
10	Tb7.NT.18.1	1	5	VYWNGESGDVFVGSFIVAKGITERK
10	Tb7.NT.18.1	1	6	APVAPQQPVPSKSVQGPRGNSAAR
10	Tb7.NT.18.1	1	6	CLTEKVMSMFVAYDEFYSGEVPLSIAFEILEYCGLVTQTVGKFK
10	Tb7.NT.18.1	1	6	DSEWMTAEVLQAVSVGKANKGGFK
10	Tb7.NT.18.1	1	6	IASDEANSPADIVEAPISVIPSRNLTR
10	Tb7.NT.18.1	1	6	LLNPTVKGVTDATSFLYELAFGSVFNPK
10	Tb7.NT.18.1	1	6	MPGQSYSSGGDYTTTVKAAAPARPVEPPKPETHSHTAPPAASPIVR
10	Tb7.NT.18.1	1	6	MPGQSYSSGGDYTTTVKAAAPARPVEPPKPETHSHTAPPAASPIVRGR
10	Tb7.NT.18.1	1	6	SGDYSIELSGEGGVTSSESTEIPSPARIR
10	Tb7.NT.18.1	1	6	TILLAAPSEAEPRFHSLPRGCIVPHSSMEDTLSSFLDQLAELQVETMER
10	Tb7.NT.18.1	1	6	VMSMFVAYDEFYSGEVPLSIAFEILEYCGLVTQTVGKFK
10	Tb7.NT.18.1	1	6	VSDSQRIASDEANSPADIVEAPISVIPSR
10	Tb7.NT.18.1	1	7	APFMINGDKDDELNTLLMSTANSYTTVSANIWTQK
10	Tb7.NT.18.1	1	7	CLSLHRHVSLMSLMCAAVISGRDGGSHPLR
10	Tb7.NT.18.1	1	7	CLTEKVMSMFVAYDEFYSGEVPLSIAFEILEYCGLVTQTVGK
10	Tb7.NT.18.1	1	7	DDELNTLLMSTANSYTTVSANIWTQK
10	Tb7.NT.18.1	1	7	IFLRPQVSQGQILLCVADQPQEATENIVDDAEGEK
10	Tb7.NT.18.1	1	7	KVSDSQRIASDEANSPADIVEAPISVIPSR
10	Tb7.NT.18.1	1	7	LLANNSNLRDRYLCEGDDAVLHDTIRPK
10	Tb7.NT.18.1	1	7	STVETTTSLIKPTTSGYTGSESYSASK
10	Tb7.NT.18.1	1	7	VSDSQRIASDEANSPADIVEAPISVIPSRNLTR
10	Tb7.NT.18.1	1	7	VSTESYTGSYSSYSYSYSDSTDEGHNVVASARSGHVAGAAQLDSEKK
10	Tb7.NT.18.1	1	8	CLSLHRHVSLMSLMCAAVISGR
10	Tb7.NT.18.1	1	8	DDELNTLLMSTANSYTTVSANIWTQKGVEGCYLEVGVEKLR
10	Tb7.NT.18.1	1	8	GRVQDDDSYSYSYSYSYGSVSSPEVK
10	Tb7.NT.18.1	1	8	HVSLMSLMCAAVISGRDGGSHPLRWLR
10	Tb7.NT.18.1	1	8	KVAVQLLRYIEAFLYVCHAIMVVTVGTGMK
10	Tb7.NT.18.1	1	8	QQPPTGSRRPSLRTPEIPK
10	Tb7.NT.18.1	1	8	TQQMPSKTQLGRAPATSTEGLK
10	Tb7.NT.18.1	1	9	FHSLPRGCIVPHSSMEDTLSSFLDQLAELQVETMER
10	Tb7.NT.18.1	1	9	FHSLPRGCIVPHSSMEDTLSSFLDQLAELQVETMERNR
11	Tb9.NT.9.3	1	2	MEDDPVYAALFTAFPQNNKNSTKVEDGSQNHDTK
12	Tb9.NT.98.1	1	10	AMVPKLVPRESMFLLDLPK
12	Tb9.NT.98.1	1	10	EISSLSKDVDVLSVKGGEVGSVDTR
12	Tb9.NT.98.1	1	10	ETILTNLIQQSMDKAVSDNAQK
12	Tb9.NT.98.1	1	10	LSIQATEPMHQQPGDALSAEQTLLNNAGENTVLK
12	Tb9.NT.98.1	1	10	SETSGLYEPHSEGSAFMSTQPGPGQLGRGEYLQPHPPPQRK
12	Tb9.NT.98.1	1	10	VATGSERPNQAGRNLHSGRDTPSTYSADLLEPYSRPLSR
12	Tb9.NT.98.1	1	11	EIVQGASRSLSPQQQVPNSWAPTLQSAMRAAPSGR
12	Tb9.NT.98.1	1	11	GAYTLLTPDNVRHVGNLYKIAVVAQDR
12	Tb9.NT.98.1	1	11	IASRVANAHYHCTPVGPTPSRFSMTR

12	Tb9.NT.98.1	1	11	NEVYMGGAAGDEGETGAIVSKVLNK
12	Tb9.NT.98.1	1	11	NLHSGRDTPSTYSADLLEPYSRPLSR
12	Tb9.NT.98.1	1	11	NQSAASPSVVFSCDGVLHDTKEENTGR
12	Tb9.NT.98.1	1	11	RPWVVDGGSPCSLRPGAGRSESLGSPALRTSSR
12	Tb9.NT.98.1	1	11	TINKQQHLDTNGEQEGKETILTNLIQQSMDK
12	Tb9.NT.98.1	1	12	AEGVSTERVGSTISMSGDIANVSDKEAGGVSALPDVSSSMR
12	Tb9.NT.98.1	1	12	AHPGDGHLDGRVATGSERPNQAGR
12	Tb9.NT.98.1	1	12	DIAHSLGVPHGNVSVALNCRNSNMVEVSIQHDGK
12	Tb9.NT.98.1	1	12	HVGNLYKIAVVAQDRNTQGNQR
12	Tb9.NT.98.1	1	12	QSGDISLQSTYDGTWGKGGYPR
12	Tb9.NT.98.1	1	12	TFDTSRWTGTPSPGAVSSSQMEAK
12	Tb9.NT.98.1	1	13	DIITTCELNNVHKLYELLEGKR
12	Tb9.NT.98.1	1	13	RCQTAEANNNADDSPKAEGVSTER
12	Tb9.NT.98.1	1	14	DTPSTYSADLLEPYSRPLSRFEKQSGDISLQSTYDGTWGK
12	Tb9.NT.98.1	1	14	NAELLQLSVVFITYFDKNKGAYTLLTPDNVR
12	Tb9.NT.98.1	1	14	TFDTSRWTGTPSPGAVSSSQMEAKGGCPGFAATCSSQR
12	Tb9.NT.98.1	1	14	YFTSRAHPGDGHLDGRVATGSERPNQAGR
12	Tb9.NT.98.1	1	15	NSNMVEVSIQHDGKLSDAAIRDIITTCELNNVHK
12	Tb9.NT.98.1	1	16	DTPSTYSADLLEPYSRPLSRFEK
12	Tb9.NT.98.1	1	16	SSSAGDAAEAWEEMSQDMLDDIQYVAFEETLNR
12	Tb9.NT.98.1	1	17	FTSRAHPGDGHLDGRVATGSERPNQAGR
12	Tb9.NT.98.1	1	17	LTLDNSGVEGKVFRFEADACNEMR
12	Tb9.NT.98.1	1	17	YDALGDTFSLTATLACSSPRKPCLLSQRSTPSPLSEMSPK
12	Tb9.NT.98.1	1	18	EENTGRNVSLQSAEAENRRPWVVDGGSPCSLRPGAGR
12	Tb9.NT.98.1	1	19	FEKQSGDISLQSTYDGTWGKGGYPR
12	Tb9.NT.98.1	1	1	DGWNRLSPGSPSHHSSSWGPPYSPTGSNALSTTVDSTSQTARR
12	Tb9.NT.98.1	1	1	DQLTPVGKCQNIDEKER
12	Tb9.NT.98.1	1	1	EDAAIPTADVDAKELSKEDGGSDGFVCDGEGSK
12	Tb9.NT.98.1	1	1	EDGGSDGFVCDGEGSKTDVTGTAAPEDVEDFEIDGVPSVGSDVSSANRLTLDNSGVEGK
12	Tb9.NT.98.1	1	1	EKNQSAASPSVVFSCDGVLHDTK
12	Tb9.NT.98.1	1	1	EPPTSQPFWEEHSSSVTTEYLHGHLGDVRTATEVETNKGNQQQQTLHTHK
12	Tb9.NT.98.1	1	1	EQDTGKQTGGTTPTNLEHKINSSNR
12	Tb9.NT.98.1	1	1	EVMSLRLALQQYQQPPCQATEQQFNGCQGVNTSQLLINNGNCQEHTTVTR
12	Tb9.NT.98.1	1	1	GNQQQQTLHTHKVEQVFAPQVNR
12	Tb9.NT.98.1	1	1	LSIQATEPMHQQPGDALSAEQTLLNNAGENTVLKGSTGSEGGTPSLNDTVATQEIVPSRR
12	Tb9.NT.98.1	1	1	LSPGSPSHHSSSWGPPYSPTGSNALSTTVDSTSQTARR
12	Tb9.NT.98.1	1	1	MPSPVFTQQSYQDSTSTLTERDGWNRLSPGSPSHHSSSWGPPYSPTGSNALSTTVDSTSQTAR
12	Tb9.NT.98.1	1	1	NSAVDAARDAVITTLNNALGSGCASAGVSSKEK
12	Tb9.NT.98.1	1	1	QDAAIPTADDDAKELSKQDAAIPTADDDAK
12	Tb9.NT.98.1	1	1	QEGTETCDDFTTKETNNNQQEKPLTTHENEHTVHNQFDSPQHGKTINK
12	Tb9.NT.98.1	1	1	QNTSLRVVPFHLCQAYEK
12	Tb9.NT.98.1	1	1	QTGGTTPTNLEHKINSSNRLSIQATEPMHQQPGDALSAEQTLLNNAGENTVLK
12	Tb9.NT.98.1	1	1	SLSPQQQVPNSWAPTLQSAMR
12	Tb9.NT.98.1	1	1	SVPSAVEHETNVCSEEQFAGTTATESSEQYIMSATTAIQKEVMSLR
12	Tb9.NT.98.1	1	1	TDVTGTAAPEDVEDFEIDGVPSVGSDVSSANR
12	Tb9.NT.98.1	1	1	TDVTGTAAPEDVEDFEIDGVPSVGSDVSSANRLTLDNSGVEGK
12	Tb9.NT.98.1	1	1	VANAHYHCTPVGPTPSRFSMTR
12	Tb9.NT.98.1	1	1	VLGFNEDALVPGNEKSVPSAVEHETNVCSEEQFAGTTATESSEQYIMSATTAIQK
12	Tb9.NT.98.1	1	1	VLGFNEDALVPGNEKSVPSAVEHETNVCSEEQFAGTTATESSEQYIMSATTAIQKEVMSLR
12	Tb9.NT.98.1	1	1	VVPFHLCQAYEKDIAHSLGVPHGNVSVALNCRNSNMVEVSIQHDGK
12	Tb9.NT.98.1	1	1	YDALGDTFSLTATLACSSPR
12	Tb9.NT.98.1	1	20	EGSPQRNEVYMGGAAGDEGETGAIVSKVLNK
12	Tb9.NT.98.1	1	21	LKYDALGDTFSLTATLACSSPRKPCLLSQR
12	Tb9.NT.98.1	1	22	CQTAEANNNADDSPKAEGVSTERVGSTISMSGDIANVSDK
12	Tb9.NT.98.1	1	22	ETNNNQQEKPLTTHENEHTVHNQFDSPQHGKTINK
12	Tb9.NT.98.1	1	23	QQHLDTNGEQEGKETILTNLIQQSMDKAVSDNAQK
12	Tb9.NT.98.1	1	23	QSGDISLQSTYDGTWGKGGYPRSQGASGSR
12	Tb9.NT.98.1	1	24	NLHSGRDTPSTYSADLLEPYSRPLSRFEK
12	Tb9.NT.98.1	1	26	KEGSPQRNEVYMGGAAGDEGETGAIVSK
12	Tb9.NT.98.1	1	28	ESPNQRHAEQELANGMQPTTIVEGEER
12	Tb9.NT.98.1	1	2	AEGVSTERVGSTISMSGDIANVSDK
12	Tb9.NT.98.1	1	2	DIAHSLGVPHGNVSVALNCRNSNMVEVSIQHDGKLSDAAIR
12	Tb9.NT.98.1	1	2	EAGGVSALPDVSSSMRSHSKLSGTPSR
12	Tb9.NT.98.1	1	2	EDAAIPTADVDAKELSKEDAAIPTADVDAK
12	Tb9.NT.98.1	1	2	EIVQGASRSLSPQQQVPNSWAPTLQSAMR
12	Tb9.NT.98.1	1	2	EPPTSQPFWEEHSSSVTTEYLHGHLGDVR
12	Tb9.NT.98.1	1	2	GGYPRSQGASGSRLEGTINGENWSLEFISDSGELVGSMLVGAPHVDGPLTIETVLETIQK
12	Tb9.NT.98.1	1	2	IKQEGTETCDDFTTKETNNNQQEKPLTTHENEHTVHNQFDSPQHGK
12	Tb9.NT.98.1	1	2	KPCLLSQRSTPSPLSEMSPK
12	Tb9.NT.98.1	1	2	LGRIASRVANAHYHCTPVGPTPSR
12	Tb9.NT.98.1	1	2	LNLAQDTGNSEPFGPDFQGERLVYLGLYPDLGFGDGVNLRPPR
12	Tb9.NT.98.1	1	2	LSPGSPSHHSSSWGPPYSPTGSNALSTTVDSTSQTARRLGR
12	Tb9.NT.98.1	1	2	NAELLQLSVVFITYFDKNK
12	Tb9.NT.98.1	1	2	NEVYMGGAAGDEGETGAIVSK
12	Tb9.NT.98.1	1	2	NEVYMGGAAGDEGETGAIVSKVLNKLNSTGK
12	Tb9.NT.98.1	1	2	RPWVVDGGSPCSLRPGAGRSESLGSPALR
12	Tb9.NT.98.1	1	2	SLSPQQQVPNSWAPTLQSAMRAAPSGRMPSPVFTQQSYQDSTSTLTER
12	Tb9.NT.98.1	1	2	SVPSAVEHETNVCSEEQFAGTTATESSEQYIMSATTAIQK
12	Tb9.NT.98.1	1	2	TATEVETNKGNQQQQTLHTHKVEQVFAPQVNR
12	Tb9.NT.98.1	1	2	VTLPPVAEVRSETSGLYEPHSEGSAFMSTQPGPGQLGRGEYLQPHPPPQR

12	Tb9.NT.98.1	1	30	MYFTSRAHPGDGHLDGRVATGSERPNQAGR
12	Tb9.NT.98.1	1	31	EGSPQRNEVYMGGAAGDEGETGAIVSK
12	Tb9.NT.98.1	1	32	QTRESPNQRHAEQELANGMQPTTIVEGEER
12	Tb9.NT.98.1	1	3	AAPSGRMPSPVFTQQSYQDSTSTLTER
12	Tb9.NT.98.1	1	3	DIITTCELNNVHKLYELLEGK
12	Tb9.NT.98.1	1	3	EDGGSDGFVCDGEGSKTDVTGTAAPEDVEDFEIDGVPSVGSDVSSANR
12	Tb9.NT.98.1	1	3	ELSKQDAAIPTADDDAKELSK
12	Tb9.NT.98.1	1	3	ETILTNLIQQSMDKAVSDNAQKVIDNITITELQQK
12	Tb9.NT.98.1	1	3	FEADACNEMREISSLSKDVDVLSVK
12	Tb9.NT.98.1	1	3	GSTGSEGGTPSLNDTVATQEIVPSR
12	Tb9.NT.98.1	1	3	GSTGSEGGTPSLNDTVATQEIVPSRRASDK
12	Tb9.NT.98.1	1	3	HAEQELANGMQPTTIVEGEERTSKEQDTGK
12	Tb9.NT.98.1	1	3	IAVVAQDRNTQGNQRSGSDR
12	Tb9.NT.98.1	1	3	KLNLAQDTGNSEPFGPDFQGERLVYLGLYPDLGFGDGVNLRPPR
12	Tb9.NT.98.1	1	3	LKYDALGDTFSLTATLACSSPR
12	Tb9.NT.98.1	1	3	LNLAQDTGNSEPFGPDFQGERLVYLGLYPDLGFGDGVNLRPPRLPAK
12	Tb9.NT.98.1	1	3	NKGAYTLLTPDNVRHVGNLYK
12	Tb9.NT.98.1	1	3	NQSAASPSVVFSCDGVLHDTKEENTGRNVSLQSAEAENR
12	Tb9.NT.98.1	1	3	SESLGSPALRTSSRFTPSVTGERPASSISCLR
12	Tb9.NT.98.1	1	3	SKQNTSLRVVPFHLCQAYEK
12	Tb9.NT.98.1	1	3	VANAHYHCTPVGPTPSRFSMTRVQPVASR
12	Tb9.NT.98.1	1	3	VGSTISMSGDIANVSDKEAGGVSALPDVSSSMR
12	Tb9.NT.98.1	1	3	VTLPPVAEVRSETSGLYEPHSEGSAFMSTQPGPGQLGR
12	Tb9.NT.98.1	1	4	AHPGDGHLDGRVATGSERPNQAGRNLHSGR
12	Tb9.NT.98.1	1	4	AVSDNAQKVIDNITITELQQK
12	Tb9.NT.98.1	1	4	DTPSTYSADLLEPYSRPLSR
12	Tb9.NT.98.1	1	4	ESMFLLDLPKSKQNTSLR
12	Tb9.NT.98.1	1	4	HAEQELANGMQPTTIVEGEER
12	Tb9.NT.98.1	1	4	SQVQSVKTKEPPTSQPFWEEHSSSVTTEYLHGHLGDVR
12	Tb9.NT.98.1	1	4	SSSAGDAAEAWEEMSQDMLDDIQYVAFEETLNREALYTAEQHIRYK
12	Tb9.NT.98.1	1	5	DLQHCVLNARDVGSSPFDYREK
12	Tb9.NT.98.1	1	5	ETNNNQQEKPLTTHENEHTVHNQFDSPQHGK
12	Tb9.NT.98.1	1	5	FEKQSGDISLQSTYDGTWGK
12	Tb9.NT.98.1	1	5	GAYTLLTPDNVRHVGNLYK
12	Tb9.NT.98.1	1	5	GGCPGFAATCSSQRSSSAGDAAEAWEEMSQDMLDDIQYVAFEETLNR
12	Tb9.NT.98.1	1	5	GGKLTSFSSAVVEPVVFGEAIVVSVDVCAQLVVDGEEFIR
12	Tb9.NT.98.1	1	5	LEGTINGENWSLEFISDSGELVGSMLVGAPHVDGPLTIETVLETIQK
12	Tb9.NT.98.1	1	5	RLKYDALGDTFSLTATLACSSPR
12	Tb9.NT.98.1	1	5	SETSGLYEPHSEGSAFMSTQPGPGQLGR
12	Tb9.NT.98.1	1	5	SSSAGDAAEAWEEMSQDMLDDIQYVAFEETLNREALYTAEQHIR
12	Tb9.NT.98.1	1	5	TKEPPTSQPFWEEHSSSVTTEYLHGHLGDVR
12	Tb9.NT.98.1	1	6	DAVITTLNNALGSGCASAGVSSKEKTAR
12	Tb9.NT.98.1	1	6	GSTGSEGGTPSLNDTVATQEIVPSRR
12	Tb9.NT.98.1	1	6	QQHLDTNGEQEGKETILTNLIQQSMDK
12	Tb9.NT.98.1	1	6	QRVTLPPVAEVRSETSGLYEPHSEGSAFMSTQPGPGQLGR
12	Tb9.NT.98.1	1	6	SLSPQQQVPNSWAPTLQSAMRAAPSGR
12	Tb9.NT.98.1	1	6	TSSRFTPSVTGERPASSISCLRAVAAR
12	Tb9.NT.98.1	1	7	DVGSSPFDYREKNQSAASPSVVFSCDGVLHDTK
12	Tb9.NT.98.1	1	7	ELSKEDGGSDGFVCDGEGSKTDVTGTAAPEDVEDFEIDGVPSVGSDVSSANR
12	Tb9.NT.98.1	1	7	ETNNNQQEKPLTTHENEHTVHNQFDSPQHGKTINKQQHLDTNGEQEGK
12	Tb9.NT.98.1	1	7	HAEQELANGMQPTTIVEGEERTSK
12	Tb9.NT.98.1	1	7	NSNMVEVSIQHDGKLSDAAIR
12	Tb9.NT.98.1	1	7	TDVTGTAAPEDVEDFEIDGVPSVGSDVSSANRLTLDNSGVEGKVFR
12	Tb9.NT.98.1	1	7	TKEPPTSQPFWEEHSSSVTTEYLHGHLGDVRTATEVETNK
12	Tb9.NT.98.1	1	7	VEQVFAPQVNRVLGFNEDALVPGNEK
12	Tb9.NT.98.1	1	8	AVSDNAQKVIDNITITELQQKNK
12	Tb9.NT.98.1	1	8	LSDAAIRDIITTCELNNVHKLYELLEGK
12	Tb9.NT.98.1	1	8	QNTSLRVVPFHLCQAYEKDIAHSLGVPHGNVSVALNCR
12	Tb9.NT.98.1	1	8	TNNDAAHDAKELSKQDAAIPTADDDAK
12	Tb9.NT.98.1	1	8	VGSTISMSGDIANVSDKEAGGVSALPDVSSSMRSHSK
12	Tb9.NT.98.1	1	8	WSSEEMSNMIYDGAPTGGGVSAVRSTLR
12	Tb9.NT.98.1	1	9	ESPNQRHAEQELANGMQPTTIVEGEERTSK
12	Tb9.NT.98.1	1	9	KPCLLSQRSTPSPLSEMSPKK
12	Tb9.NT.98.1	1	9	VVPFHLCQAYEKDIAHSLGVPHGNVSVALNCR
13	Tb1.NT.38.9	1	10	AEDLDSYRPVTLTSCLCKVMERIIAAR
13	Tb1.NT.38.9	1	10	DQQRLAGTPFFTCRSLGTCQR
13	Tb1.NT.38.9	1	10	GGGVSILVREDLPVETGIAVVGRIEQVHAIIR
13	Tb1.NT.38.9	1	10	GVPQGTVLGPIMFIIVMNSLSQRLAEVPLLQHGFFADDLTLLAR
13	Tb1.NT.38.9	1	10	MPATSAWCQGPVPRIIGGSQEPAEFLSWGTLLCSGYGIIQHR
13	Tb1.NT.38.9	1	11	CTECDATYQCRSSAVTHMVNKHGFVR
13	Tb1.NT.38.9	1	11	LAGTPFFTCRSLGTCQRAISSIIR
13	Tb1.NT.38.9	1	12	AISSIIRTKILLSGDVEENPGPSLR
13	Tb1.NT.38.9	1	12	GVPQGTVLGPIMFIIVMNSLSQR
13	Tb1.NT.38.9	1	12	HSCRPIFHTQIKPVCADDPDDVK
13	Tb1.NT.38.9	1	12	LFNESLRTGVVPPAWKTGVIIPILK
13	Tb1.NT.38.9	1	12	LLGVTFQCLQGMATHAAETRR
13	Tb1.NT.38.9	1	12	RHPDSHPPAPIKTIASEFSPITMAELR
13	Tb1.NT.38.9	1	12	STPSRLSIFSDSLSMLTALQTGPLAVTDPILRR
13	Tb1.NT.38.9	1	13	ATPPHWTHTLPKLDEEIAGCGPSHRR
13	Tb1.NT.38.9	1	13	HTEGDVINHTLQCGLNVVLQWSKEYFMSVNVAKTK

13	Tb1.NT.38.9	1	13	SCHIVKKVYAPRPLTTPAVLVDNAAITDYR
13	Tb1.NT.38.9	1	14	GCRATPPHWTHTLPKLDEEIAGCGPSHR
13	Tb1.NT.38.9	1	14	IASCLLSETRMTPGEAACFSVAGYQHHGIAR
13	Tb1.NT.38.9	1	14	RQVLRAFYLALAQAHTMYGIEVWYWDASER
13	Tb1.NT.38.9	1	15	MTPGEAACFSVAGYQHHGIARNCKGGGVSILVR
13	Tb1.NT.38.9	1	16	EHYELWTDGSVSLGEKSGSAALLYRNNTLICAPK
13	Tb1.NT.38.9	1	16	ILLSGDVEENPGPSLRGMQWNCAGLSQR
13	Tb1.NT.38.9	1	18	EYFMSVNVAKTKCTLFGCIER
13	Tb1.NT.38.9	1	18	IASCLLSETRMTPGEAACFSVAGYQHHGIARNCK
13	Tb1.NT.38.9	1	19	EDLPVETGIAVVGRIEQVHAIIRLAR
13	Tb1.NT.38.9	1	1	ATSAWCQGPVPRIIGGSQEPAEFLSWGTLLCSGYGIIQHR
13	Tb1.NT.38.9	1	1	GTALTVTSAYIPPKHTFTATDLYTLLTTDGAQLIGADANAHALSWDRASPPNTK
13	Tb1.NT.38.9	1	1	HHGESTPDVTLSRNCTVYTWTSLYSPDSDHHHTFFDVIVGDDTDALSCPRLR
13	Tb1.NT.38.9	1	1	IIGGSQEPAEFLSWGTLLCSGYGIIQHRDQQRLAGTPFFTCR
13	Tb1.NT.38.9	1	1	INPSVPPQCRWCNPQHAAIGPTIQTAPPVATR
13	Tb1.NT.38.9	1	1	LLPSGSAAEPDCLYNEALQHLGR
13	Tb1.NT.38.9	1	1	MTPGEAACFSVAGYQHHGIAR
13	Tb1.NT.38.9	1	1	NCTVYTWTSLYSPDSDHHHTFFDVIVGDDTDALSCPRLRKPMYAWLK
13	Tb1.NT.38.9	1	1	NLEILSAAQHKASRIIAGIPHGTR
13	Tb1.NT.38.9	1	1	RSIKLLPSGSAAEPDCLYNEALQHLGR
13	Tb1.NT.38.9	1	1	SGEVHENTHRFGITGNHFPTK
13	Tb1.NT.38.9	1	1	SGSAALLYRNNTLICAPKTGEGELSCSYR
13	Tb1.NT.38.9	1	1	TGVVPPAWKTGVIIPILKAGK
13	Tb1.NT.38.9	1	1	VPMRPQVLHCTLLCLQIR
13	Tb1.NT.38.9	1	1	VYAPRPLTTPAVLVDNAAITDYRQAERFSK
13	Tb1.NT.38.9	1	20	CTLFGCIERHPLTLQLDGERIGADR
13	Tb1.NT.38.9	1	20	KVYAPRPLTTPAVLVDNAAITDYRQAER
13	Tb1.NT.38.9	1	22	HSCRPIFHTQIKPVCADDPDDVKR
13	Tb1.NT.38.9	1	22	ILLSGDVEENPGPSLRGMQWNCAGLSQRK
13	Tb1.NT.38.9	1	25	KAEDLDSYRPVTLTSCLCKVMER
13	Tb1.NT.38.9	1	26	SRTFERGVPQGTVLGPIMFIIVMNSLSQR
13	Tb1.NT.38.9	1	28	TKILLSGDVEENPGPSLRGMQWNCAGLSQR
13	Tb1.NT.38.9	1	2	AEDLDSYRPVTLTSCLCKVMER
13	Tb1.NT.38.9	1	2	AGKKAEDLDSYRPVTLTSCLCK
13	Tb1.NT.38.9	1	2	EHYELWTDGSVSLGEKSGSAALLYR
13	Tb1.NT.38.9	1	2	EPPRREHYELWTDGSVSLGEK
13	Tb1.NT.38.9	1	2	GETLTQWCIDNQFLVCNTCECTRYTRHHGESTPDVTLSR
13	Tb1.NT.38.9	1	2	HPLTLQLDGERIGADRTPK
13	Tb1.NT.38.9	1	2	HYEWMLRKINPSVPPQCR
13	Tb1.NT.38.9	1	2	IEPREHPLGTSTLRHSCRPIFHTQIKPVCADDPDDVK
13	Tb1.NT.38.9	1	2	LLGVTFQCLQGMATHAAETRRK
13	Tb1.NT.38.9	1	2	LLPSGSAAEPDCLYNEALQHLGRTALNVVLRLFNESLR
13	Tb1.NT.38.9	1	2	LLQIAAISASTWGPRRQVLR
13	Tb1.NT.38.9	1	2	LSIFSDSLSMLTALQTGPLAVTDPILR
13	Tb1.NT.38.9	1	2	MDFRLLQIAAISASTWGPRR
13	Tb1.NT.38.9	1	2	PATSAWCQGPVPRIIGGSQEPAEFLSWGTLLCSGYGIIQHRDQQR
13	Tb1.NT.38.9	1	2	RIKYGDATPAVDIPPEPPPVLAIVPLPSSTR
13	Tb1.NT.38.9	1	2	STPSRLSIFSDSLSMLTALQTGPLAVTDPILR
13	Tb1.NT.38.9	1	2	TALNVVLRLFNESLRTGVVPPAWK
13	Tb1.NT.38.9	1	2	TFERGVPQGTVLGPIMFIIVMNSLSQR
13	Tb1.NT.38.9	1	2	TPKLLGVTFQCLQGMATHAAETRR
13	Tb1.NT.38.9	1	2	TVQRTSESTKCTECDATYQCR
13	Tb1.NT.38.9	1	2	VYAPRPLTTPAVLVDNAAITDYR
13	Tb1.NT.38.9	1	2	YGDATPAVDIPPEPPPVLAIVPLPSSTRVPMRPQVLHCTLLCLQIR
13	Tb1.NT.38.9	1	36	IGADRTPKLLGVTFQCLQGMATHAAETR
13	Tb1.NT.38.9	1	3	CTLFGCIERHPLTLQLDGER
13	Tb1.NT.38.9	1	3	DTVESQLTPQQSGFRPGCSTLEQLLHVRAALCRPTHQSRTGAVFVDYEK
13	Tb1.NT.38.9	1	3	EHPLGTSTLRHSCRPIFHTQIKPVCADDPDDVK
13	Tb1.NT.38.9	1	3	GGGVSILVREDLPVETGIAVVGR
13	Tb1.NT.38.9	1	3	HSCRPIFHTQIKPVCADDPDDVKREASEK
13	Tb1.NT.38.9	1	3	HTFTATDLYTLLTTDGAQLIGADANAHALSWDR
13	Tb1.NT.38.9	1	3	HTFTATDLYTLLTTDGAQLIGADANAHALSWDRASPPNTK
13	Tb1.NT.38.9	1	3	KINPSVPPQCRWCNPQHAAIGPTIQTAPPVATR
13	Tb1.NT.38.9	1	3	LRDTVESQLTPQQSGFRPGCSTLEQLLHVRAALCRPTHQSR
13	Tb1.NT.38.9	1	3	NCTVYTWTSLYSPDSDHHHTFFDVIVGDDTDALSCPR
13	Tb1.NT.38.9	1	3	NCTVYTWTSLYSPDSDHHHTFFDVIVGDDTDALSCPRLR
13	Tb1.NT.38.9	1	3	TSESTKCTECDATYQCRSSAVTHMVNK
13	Tb1.NT.38.9	1	4	AADLPQLRDTWIPDIIAYAKR
13	Tb1.NT.38.9	1	4	AFYLALAQAHTMYGIEVWYWDASERNLEILSAAQHK
13	Tb1.NT.38.9	1	4	DTVESQLTPQQSGFRPGCSTLEQLLHVRAALCRPTHQSR
13	Tb1.NT.38.9	1	4	HTEGDVINHTLQCGLNVVLQWSKEYFMSVNVAK
13	Tb1.NT.38.9	1	4	IIGGSQEPAEFLSWGTLLCSGYGIIQHR
13	Tb1.NT.38.9	1	4	KVYAPRPLTTPAVLVDNAAITDYR
13	Tb1.NT.38.9	1	4	LLGVTFQCLQGMATHAAETR
13	Tb1.NT.38.9	1	4	LSIFSDSLSMLTALQTGPLAVTDPILRR
13	Tb1.NT.38.9	1	4	NNTLICAPKTGEGELSCSYRAECVALEIGLQR
13	Tb1.NT.38.9	1	4	QVLRAFYLALAQAHTMYGIEVWYWDASERNLEILSAAQHK
13	Tb1.NT.38.9	1	4	REHYELWTDGSVSLGEKSGSAALLYR
13	Tb1.NT.38.9	1	4	SSAVTHMVNKHGFVRADALR
13	Tb1.NT.38.9	1	4	SYPHLRIEPREHPLGTSTLR

13	Tb1.NT.38.9	1	4	VSVPRGCRATPPHWTHTLPK
13	Tb1.NT.38.9	1	5	ASPPNTKGETLTQWCIDNQFLVCNTCECTRYTR
13	Tb1.NT.38.9	1	5	ATPPHWTHTLPKLDEEIAGCGPSHR
13	Tb1.NT.38.9	1	5	GETLTQWCIDNQFLVCNTCECTR
13	Tb1.NT.38.9	1	5	IIAARLRDTVESQLTPQQSGFRPGCSTLEQLLHVR
13	Tb1.NT.38.9	1	5	LARGTALTVTSAYIPPKHTFTATDLYTLLTTDGAQLIGADANAHALSWDR
13	Tb1.NT.38.9	1	5	SGEVHENTHRFGITGNHFPTKHK
13	Tb1.NT.38.9	1	5	TGEGELSCSYRAECVALEIGLQRLLK
13	Tb1.NT.38.9	1	6	AALCRPTHQSRTGAVFVDYEK
13	Tb1.NT.38.9	1	6	ATSAWCQGPVPRIIGGSQEPAEFLSWGTLLCSGYGIIQHRDQQR
13	Tb1.NT.38.9	1	6	EDLPVETGIAVVGRIEQVHAIIR
13	Tb1.NT.38.9	1	6	EHPLGTSTLRHSCRPIFHTQIKPVCADDPDDVKR
13	Tb1.NT.38.9	1	6	HPDSHPPAPIKTIASEFSPITMAELR
13	Tb1.NT.38.9	1	6	LAEVPLLQHGFFADDLTLLAR
13	Tb1.NT.38.9	1	6	NCKGGGVSILVREDLPVETGIAVVGR
13	Tb1.NT.38.9	1	6	PATSAWCQGPVPRIIGGSQEPAEFLSWGTLLCSGYGIIQHR
13	Tb1.NT.38.9	1	6	SIKLLPSGSAAEPDCLYNEALQHLGRTALNVVLR
13	Tb1.NT.38.9	1	6	TGEGELSCSYRAECVALEIGLQR
13	Tb1.NT.38.9	1	6	TLVDERIASCLLSETRMTPGEAACFSVAGYQHHGIAR
13	Tb1.NT.38.9	1	6	VSPHIVKWCVSFLSNRTGR
13	Tb1.NT.38.9	1	7	ASPPNTKGETLTQWCIDNQFLVCNTCECTR
13	Tb1.NT.38.9	1	7	LTLHKTLVDERIASCLLSETR
13	Tb1.NT.38.9	1	7	MPATSAWCQGPVPRIIGGSQEPAEFLSWGTLLCSGYGIIQHRDQQR
13	Tb1.NT.38.9	1	7	TIASEFSPITMAELRRSIK
13	Tb1.NT.38.9	1	8	AFYLALAQAHTMYGIEVWYWDASERNLEILSAAQHKASR
13	Tb1.NT.38.9	1	8	IIGGSQEPAEFLSWGTLLCSGYGIIQHRDQQR
13	Tb1.NT.38.9	1	8	MTPGEAACFSVAGYQHHGIARNCK
13	Tb1.NT.38.9	1	8	QVLRAFYLALAQAHTMYGIEVWYWDASER
13	Tb1.NT.38.9	1	8	TFERGVPQGTVLGPIMFIIVMNSLSQRLAEVPLLQHGFFADDLTLLAR
13	Tb1.NT.38.9	1	8	WLPAYRSTPSRLSIFSDSLSMLTALQTGPLAVTDPILR
13	Tb1.NT.38.9	1	9	AALCRPTHQSRTGAVFVDYEKAFDTVDHDK
13	Tb1.NT.38.9	1	9	IEQVHAIIRLARGTALTVTSAYIPPK
13	Tb1.NT.38.9	1	9	LRDTVESQLTPQQSGFRPGCSTLEQLLHVR
13	Tb1.NT.38.9	1	9	MKVSPHIVKWCVSFLSNR
13	Tb1.NT.38.9	1	9	VYAPRPLTTPAVLVDNAAITDYRQAER
14	Tb6.NT.64.8	1	10	GVPQGTVLGPIMFIIVMNSLSQRLAEVPLLQHGFFADDLTLLAR
14	Tb6.NT.64.8	1	12	GVPQGTVLGPIMFIIVMNSLSQR
14	Tb6.NT.64.8	1	12	HSCRPIFHTQIKPVCADDPDDVK
14	Tb6.NT.64.8	1	22	HSCRPIFHTQIKPVCADDPDDVKR
14	Tb6.NT.64.8	1	26	SRTFERGVPQGTVLGPIMFIIVMNSLSQR
14	Tb6.NT.64.8	1	2	HPLTLQLDGERIGADRTPK
14	Tb6.NT.64.8	1	2	LSIFSDSLSMLTALQTGPLAVTDPILR
14	Tb6.NT.64.8	1	2	TFERGVPQGTVLGPIMFIIVMNSLSQR
14	Tb6.NT.64.8	1	2	TTTLVRSMKFMLMCESR
14	Tb6.NT.64.8	1	2	WLPAYRSTSSRLSIFSDSLSMLTALQTGPLAVTDPILR
14	Tb6.NT.64.8	1	3	EHPLETSTLRHSCRPIFHTQIKPVCADDPDDVK
14	Tb6.NT.64.8	1	4	DTVESQLTPQQSGFRPGCSPLEQLLHVCAALCRPTHQYR
14	Tb6.NT.64.8	1	4	LSIFSDSLSMLTALQTGPLAVTDPILRR
14	Tb6.NT.64.8	1	6	FRDTVESQLTPQQSGFRPGCSPLEQLLHVCAALCRPTHQYR
14	Tb6.NT.64.8	1	6	LAEVPLLQHGFFADDLTLLAR
14	Tb6.NT.64.8	1	6	VSPHIVKWCVSFLSNRTGR
14	Tb6.NT.64.8	1	8	LSIFSDSLSMLTALQTGPLAVTDPILR
14	Tb6.NT.64.8	1	9	MKVSPHIVKWCVSFLSNR
15	Tb6.NT.63.6	0	4	DTVESQLTPQQSGFRPGCSPLEQLLHVCAALCRPTHQYR
15	Tb6.NT.63.6	0	6	FRDTVESQLTPQQSGFRPGCSPLEQLLHVCAALCRPTHQYR
15	Tb6.NT.63.6	0	6	LAEVPLLQHGFFADDLTLLAR
15	Tb6.NT.63.6	0	6	VSPHIVKWCVSFLSNRTGR
15	Tb6.NT.63.6	0	9	MKVSPHIVKWCVSFLSNR
16	Tb9.NT.107.4	0	4	SYPHLRIEPREHPLGTSTLR
17	Tb11.NT.21.5	0.999	1	QHFIGECRVDMHCSSYVCVGSGLIMMGRPGKELNVIPGLAHSLFSYEMELTVTQFGR
18	Tb6.NT.63.1	0.999	1	ASSVPFMGNTAVLRLRHHTAQGSAASCWDTVFHLSVHGHVPACHQQYIIHTK
19	Tb6.NT.71.1	0.999	1	AMFFGTVVTAAVDCVEVAYKTMRNLTHPHIFIFIFMLLI
20	Tb7.NT.1.5	0.999	1	MITYVTSFFSTCQSINMVIILRVVNDINDGGDKEALLLLWSCFFFVLVTEIISR
21	Tb7.NT.18.4	0.999	1	MPSTNKPLWVISILPWLNLTYKWEFSRCLHGLNR
22	Tb7.NT.66.1	0.999	1	AYSDYNKQEASTETSHNDYYEQCYGDAAFDYHEEVGEAIAEEIRINMGR
23	Tb7.NT.97.1	0.999	1	MWTWNIFEATTFYLHSPSVMQRSLLLTSRHFDPLPPHTQNLWCHLQYTCTSK
24	Tb9.NT.2.1	0.999	1	DNEAQLLGVEFVDGTRSGNIMWK
25	Tb9.NT.97.12	0.999	1	HWLNEFMTIMNMIGPRTVPCGTPRSNVLLLESFSLNLTLPVR
26	Tb4.NT.62.2	0.999	1	MDVISICFPLLQICCSSNSMKSPFSLDIPPNLWIRPKR
27	Tb8.NT.66.3	0.999	1	GVMIPRSWERPWSNCFFRVSTVCTFHLTFSAPVMK
28	Tb9.NT.11.1	0.999	1	HMRQDNRMVECVMPFVTNETLPFFTDAGGTGAK
29	Tb11.NT.216.1	0.999	1	YDVATPLFGYAMEMTGEGSFPLRKDDER
30	Tb11.NT.217.2	0.999	1	MLHCLSCIELPLKQMMMMMMAMPFSVEQWR
31	Tb5.NT.24.4	0.999	1	MLPVCVGLSMSLCTFLYTQHFNTACMFASTRCPLHGRVTFWHLDGLLFLSISTR
